# Tracheolaryngeal Squamous Cell Carcinoma with Extensive Mucosal Spread Without Metastasis in a Female

**DOI:** 10.7759/cureus.7219

**Published:** 2020-03-09

**Authors:** Rania Al Asmar, Yousef Shweihat, Catherine Adams, Haitem Mezughi, Mohamed S Suliman

**Affiliations:** 1 Internal Medicine, Marshall University, Joan C. Edwards School of Medicine, Huntington, USA; 2 Pulmonology, Marshall University, Joan C. Edwards School of Medicine, Huntington, USA

**Keywords:** tracheal cancer, squamous cell carcinoma, chemotherapy, tracheal resection, radiation therapy

## Abstract

Tracheal tumors remain one of the most interesting and challenging respiratory tumors. Usually, with the more invasive histologic subtypes, cancer has already invaded surrounding structures at the time of diagnosis. We present an unusual case of primary tracheal squamous cell carcinoma with an extensive mucosal spread at the time of diagnosis without any invasion of surrounding organs or distant metastasis. We discuss the unique features and our treatment approach to this unusual presentation. We also discuss the various epidemiologic, diagnostic and treatment aspects of upper airways tumors of the hypopharynx, larynx, and trachea that can help patients achieve more favorable outcomes.

## Introduction

Tracheal malignant tumors remain a diagnostic challenge in clinical practice, partly due to their vague clinical presentation that overlaps with many other diagnoses. It is a rare type of tumor with multiple histopathologies. It is common usually in smokers, diagnosed at an average of 60-65 years of age. Most often, it causes local invasion, extension to adjacent structures, and distant metastasis. It is quite unlikely that locally advanced disease within the trachea does not extend into the adjacent structures soon afterward.

The main dilemma with tracheal tumors is delayed diagnosis due to alternative misdiagnoses like chronic obstructive pulmonary disease (COPD), upper respiratory tract infection, or primary thyroid cancers. Another difficulty lies in its management challenges; choosing between surgery in such a critical location versus chemotherapy and radiation therapy. Often, the best initial diagnostic test is a computed tomography (CT) scan of the neck and chest, followed by definitive diagnosis with bronchoscopy and tissue biopsy. Staging is via a positron emission tomography (PET)/CT scan, usually using the tumor-node-metastasis (TNM) system.

Surgically resectable disease is for up to 5 cm of affected trachea only, to minimize postoperative debility. Prognosis is usually better for surgically resectable disease that is followed by radiotherapy, with a high five-year survival rate of up to 50% [1].

The patient we present here had an extensive mucosal spread of the disease from the infraglottic area down to the carina at the time of diagnosis, without surrounding organ invasion or metastasis.

## Case presentation

A 62-year-old female with a past medical history of hypertension, dyslipidemia, hypothyroidism, and 30 years of cigarette smoking presented with worsening shortness of breath and foreign body sensation over two days duration. Symptoms were typically worse at night and on laying flat. On exam, she had a faint stridor and was slightly tachypneic with a respiratory rate of 22 breaths per minute. CT of the neck was performed, revealing soft tissue prominence within her cervical trachea.

Thoracic surgery explored the neck due to the presence of stridor but that did not reveal any extratracheal abnormal tissue and no apparent lymph nodes in the area. She improved with steroid treatment only to relapse with the same symptoms a few days after discharge when the steroids were tapered down. After multiple examinations, and several treatments for COPD exacerbation, the patient was referred to the pulmonology clinic.

**Figure 1:**
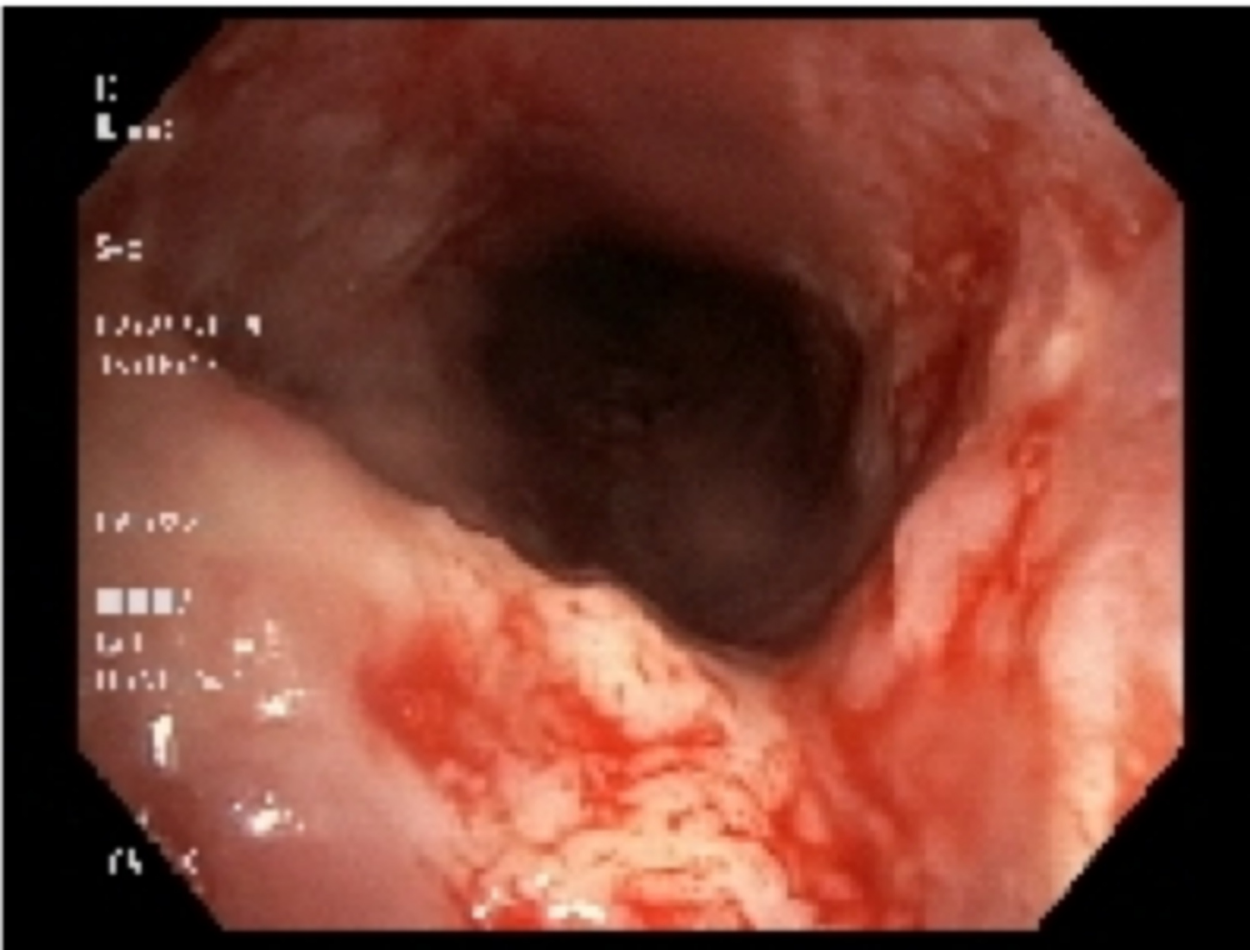
Bronchoscopic images of the mucosa of the hypopharynx and trachea studded with tumor masses, all the way down to the carina and into the proximal two mainstem bronchi.

**Figure 2:**
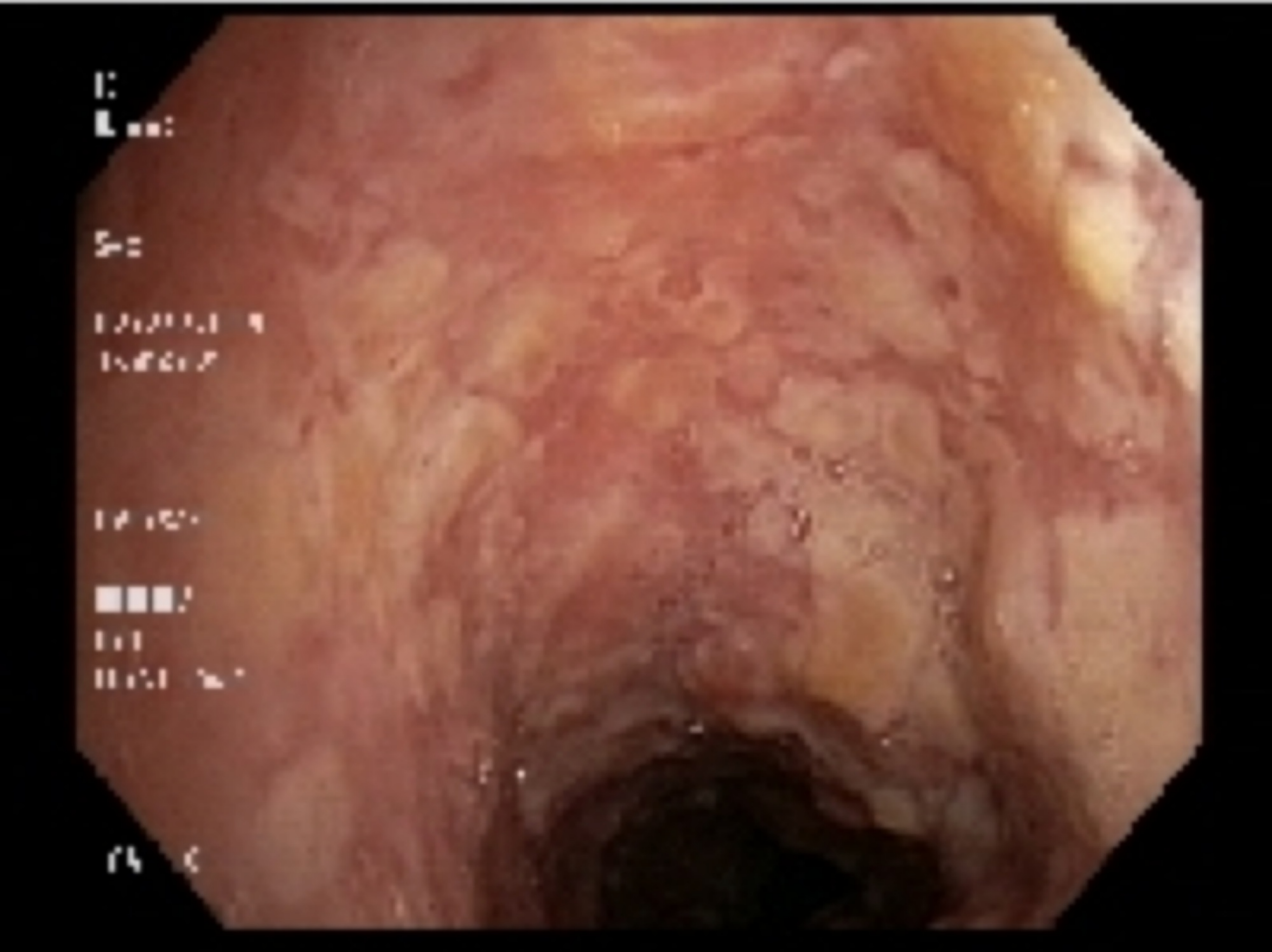
Bronchoscopic images of the mucosa of the hypopharynx and trachea studded with tumor masses, all the way down to the carina and into the proximal two mainstem bronchi.

**Figure 3:**
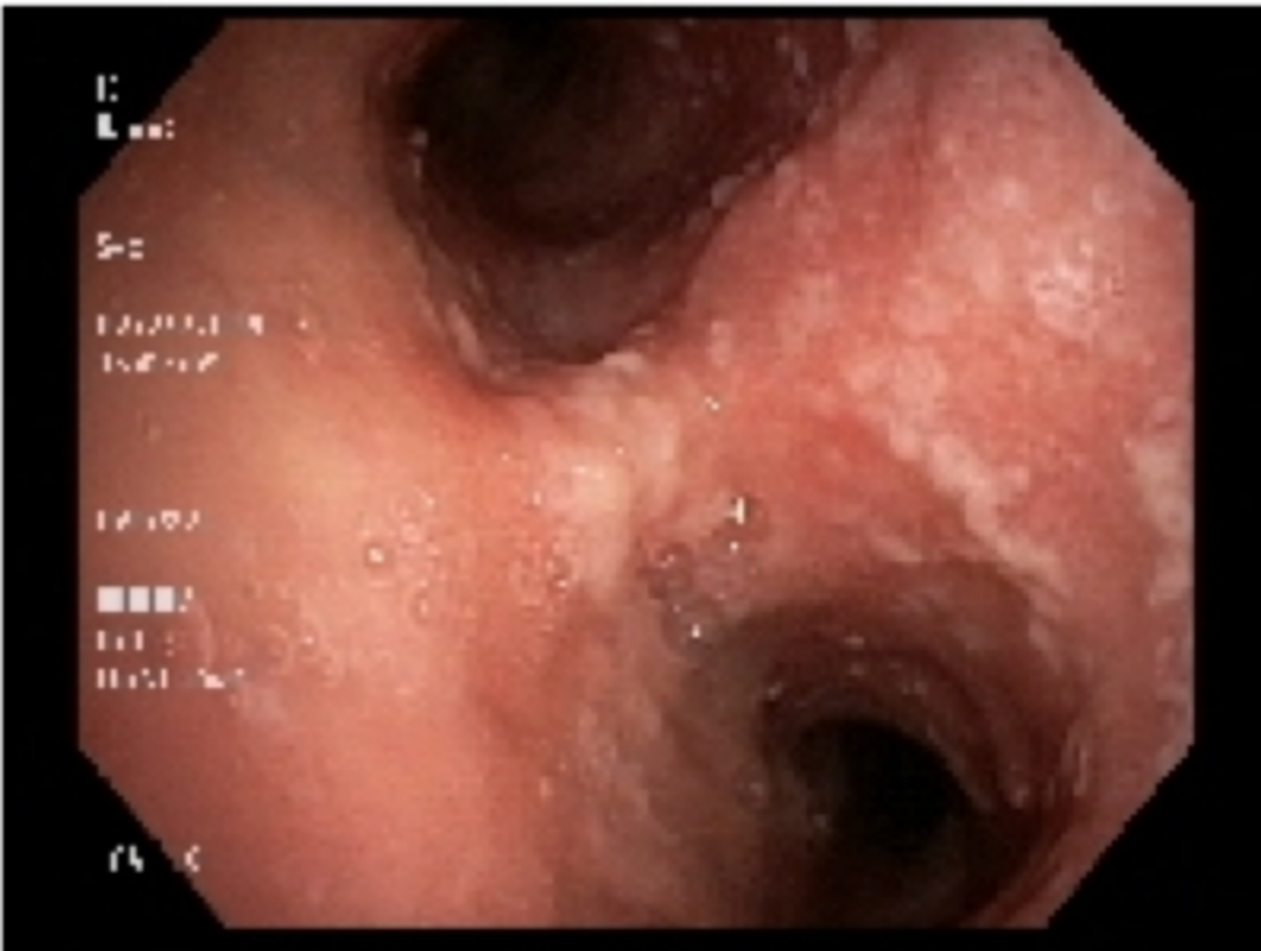
Bronchoscopic images of the mucosa of the hypopharynx and trachea studded with tumor masses, all the way down to the carina and into the proximal two mainstem bronchi.

Bronchoscopy showed that the trachea was heavily studded and infiltrated with abnormal tissue on all walls: from the vocal cords and subglottic area, down to the carina and into the proximal mainstem bronchi (Figures 1-3). Biopsy of the multiple areas showed squamous cell carcinoma (SCC) with supportive histochemistry. Her Program Death Ligand molecule 1 expression (PD-1) was low at 20%. Esophagogastroduodenoscopy (EGD) was performed next and showed normal esophageal mucosa throughout and no evidence of any fistulas. The staging process was initiated, with a PET/CT scan only showing increased uptake at the site of the locally advanced disease confined to the airways. A brain magnetic resonance imaging (MRI) was negative for metastasis. Her case was discussed in the multidisciplinary tumor board. The tumor was considered unresectable due to the apparent involvement of the entire tracheal length. Given the need to irradiate most of the mediastinum to cover all the tumor area, radiation therapy toxicity was considered too high. Thus, plans were made to re-evaluate the disease extent after two to three cycles of induction chemotherapy for possible chemo-radiation therapy if suitable at that time. The patient was initially treated with antibiotics for superimposed bacterial bronchitis, bronchodilators, and intravenous corticosteroids, which partially controlled her symptoms. Then, she was started on induction chemotherapy with cisplatin and docetaxel.

Two weeks after the second cycle of induction chemotherapy, she was readmitted with shortness of breath and stridor; repeat chest imaging revealed stable tumor size without new extension. Repeat bronchoscopy then showed worsening tracheal stenosis of mixed subtype (intrinsic and extrinsic) with tumor regression back to the carina only. Mucus plugs were removed from both lungs and then a Bonastent was placed, 16x60 mm, over the stenotic area. The patient was treated with systemic steroids and discharged home. Outpatient follow-up PET/CT showed a mild increase in the size of the tracheal disease now with cervical, supraclavicular, and mediastinal lymph nodes involvement. The patient was continued on chemotherapy and follow-up bronchoscopy to re-evaluate the stent location, and tracheal stenosis showed the distal end of the stent covered with tumor overgrowth, encroaching on the tracheal lumen, as well as granulation tissue. Repeated discussion in multidisciplinary lung tumor board resulted in a decision to start radiation therapy for local control of the disease at the tracheal stenotic level. Repeated bronchoscopic examination after finishing radiation therapy revealed regression of the tumor at the distal end of the stent. Radiologically, the tumor has regressed with continued concurrent chemo-radiotherapy to the entire tumor bed.

## Discussion

Tracheal tumors have been described in the literature for many years; however, they remain rare [2-6]. Histologically, various subtypes include but are not limited to: squamous cell carcinoma, adenoid cystic carcinoma, mucoepidermoid carcinoma, small cell carcinoma, mucosa-associated lymphoid tissue (MALT) lymphoma, chondrosarcoma, sarcomatoid carcinoma, granular cell tumor, leiomyoma, and glomus tumor [7-17]. Malignant subtypes remain far more common than benign ones [7]. Other than its wide histological variability, some tracheal tumors are interesting, as they can stay asymptomatic for so long, only incidentally found on imaging or spirometry [18]. Also, they can coexist with another type of tumor simultaneously, commonly primary thyroid cancer [9,13]. 

Due to the universal increase in smoking rates, tracheal tumors are more frequently diagnosed, together with other respiratory tumors. In November 2018, Helliwell et al. published a data set for histopathological reporting of carcinomas of the hypopharynx, larynx, and trachea in a standardized fashion. It guides pathologists on how to stage upper airway tumors based on their site, focality, dimensions (size), histological type and grade, extent of invasion, and margins for surgically treated cancers. This guide has recommended that tracheal tumors are reported like hypopharyngeal tumors, T1-T4 (T meaning tumor). As the number implies, T4 will represent tumors invading adjacent structures or organs [19].

The management of tracheal cancers remains a challenge to clinicians; surgical resection, when possible, is delicate and meticulous and can render the patient debilitated. Despite that, surgery is the most definitive modality of treatment when tumors are resectable. Also, radiation therapy to the windpipe, whether as monotherapy or following surgery, can lead to several debilities, including dysphagia, thyroid disease, and chronic dyspnea. Chemotherapy with platinum-based regimens, with and without radiation therapy, has been used for non-resectable tumors. However, recent advances in therapy are now available, including endo-bronchial laser resection, brachytherapy, and palliative stenting [20].

## Conclusions

The diagnosis and treatment of tracheal cancers in this era can be challenging to the primary care physician, oncologist, and patient. However, we report a case of early diagnosis of tracheal squamous cell carcinoma, with successful initial treatment and good patient outcomes with preserved functional capacity, including swallowing and breathing. This gives the health care team and the patients a lot of light at the end of the tunnel.
